# Engagement of Fas on Macrophages Modulates Poly I:C Induced Cytokine Production with Specific Enhancement of IP-10

**DOI:** 10.1371/journal.pone.0123635

**Published:** 2015-04-07

**Authors:** Caitriona Lyons, Philana Fernandes, Liam J. Fanning, Aileen Houston, Elizabeth Brint

**Affiliations:** 1 Department of Pathology, University College Cork, Cork, Ireland; 2 Department of Medicine, University College Cork, Cork, Ireland; 3 Alimentary Pharmabiotic Centre, University College Cork, Cork, Ireland; University of San Francisco, UNITED STATES

## Abstract

Viral double-stranded RNA (dsRNA) is recognised by pathogen recognition receptors such as Toll-Like Receptor 3 (TLR3) and retinoic acid inducible gene-I (RIG-I), and results in cytokine and interferon production. Fas, a well characterised death receptor, has recently been shown to play a role in the inflammatory response. In this study we investigated the role of Fas in the anti-viral immune response. Stimulation of Fas on macrophages did not induce significant cytokine production. However, activation of Fas modified the response of macrophages to the viral dsRNA analogue poly I:C. In particular, poly I:C-induced IP-10 production was significantly enhanced. A similar augmentation of IP-10 by Fas was observed following stimulation with both poly A:U and Sendai virus. Fas activation suppressed poly I:C-induced phosphorylation of the MAP kinases p38 and JNK, while overexpression of the Fas adaptor protein, Fas-associated protein with death domain (FADD), activated AP-1 and inhibited poly I:C-induced IP-10 production. Consistent with an inhibitory role for AP-1 in IP-10 production, mutation of the AP-1 binding site on the IP-10 promoter resulted in augmented poly I:C-induced IP-10. These results demonstrate that engagement of the Fas receptor plays a role in modifying the innate immune response to viral RNA.

## Introduction

Macrophages play a key role in the innate immune response. Detection of a viral infection by macrophages results in recruitment of a variety of immune cells to the site of infection. Initial detection of a viral infection is dependent on recognition by host Pattern Recognition Receptors (PRRs) [1]. PRRs involved in the anti-viral immune response include endosomally located Toll-Like Receptors (TLRs) 3, 7/8 and 9, which recognise viral nucleic acids such as double-stranded RNA (dsRNA), single-stranded RNA and DNA respectively [1, 2]. Signalling through TLRs 7, 8 and 9 involves recruitment of the common TLR adaptor molecule MyD88. Signalling through TLR-3 is a MyD88-independent process, utilising instead the adapter molecule TRIF [1]. Viral infection can also be detected by a group of structurally related cytosolic sensors including retinoic acid inducible gene-I (RIG-I)-like receptors, which are sensors of RNA, and AIM2-like receptors, which are sensors of DNA. Detection of dsRNA by the cytosolic sensor RIG-I results in recruitment of its adapter protein IPS-I (MAVS/Cardif/VISA) and subsequent production of anti-viral cytokines [3]. Activation of these signalling pathways results in the induction of a variety of pro-inflammatory cytokines and type I interferons (IFNs). Most cytokine activation is dependent on activation of the transcription factor NFκB whereas induction of type I IFNs is dependent on activation of IRFs 3 and 7 [1].

Macrophages also express the Fas (CD95/Apo-1) receptor, which is a well-known member of the tumour necrosis factor (TNF) family [4]. Ligation of Fas by its ligand, Fas ligand (FasL), results in the recruitment of the adaptor protein Fas-associated death domain (FADD). FADD in turn recruits the cysteine protease pro-caspase-8, resulting in the formation of the death-inducing signalling complex or DISC. Subsequent activation of effector caspases culminates in the induction of apoptosis [5]. Viruses, such as human herpes virus 8 and the poxvirus mollusc contagiosum virus, have been shown to target the Fas signalling pathway and prevent the binding of FasL to Fas [6, 7], thus protecting the infected cells from Fas-mediated apoptosis [8]. These studies highlight the importance of the apoptotic function of Fas in the host response to viral infection.

In addition to its apoptotic function, Fas has recently been shown to mediate other biological processes including cell migration, proliferation, invasion and inflammation [9]. Indeed activation of Fas in macrophages has been shown to directly induce the production of IL-1β, IL-18 and a variety of chemokines [10, 11]. Moreover, we have recently shown that extensive crosstalk exists in intestinal epithelial cells between the Fas signalling pathway and TLR4 and TLR5-mediated inflammation [12]. However, whether crosstalk also exists between Fas and the innate anti-viral immune response has not been investigated.

The aim of this study was to further characterise the role of Fas in the anti-viral innate immune response. Here we report that whilst ligation of Fas on macrophages did not induce significant cytokine production, it modified the response of macrophages to the viral dsRNA analogue poly I:C. In particular, poly I:C-induced IP-10 production was significantly enhanced. A similar augmentation of IP-10 by Fas was seen with the TLR3 ligand, poly A:U, and the RIG-I activator, Sendai virus. We demonstrate that mutation of the AP-1 binding site on the IP-10 promoter also resulted in augmented poly I:C-induced IP-10, with Fas activation suppressing poly I:C-induced phosphorylation of the MAP kinases upstream of AP-1. Moreover we have shown a role for the Fas adaptor protein FADD in the regulation of IP-10 production. These results demonstrate that engagement of the Fas receptor plays a role in modifying the innate immune system in response to viral RNA.

## Results

### Stimulation of macrophages with poly I:C induces Fas and FasL expression

**Fig 1:**
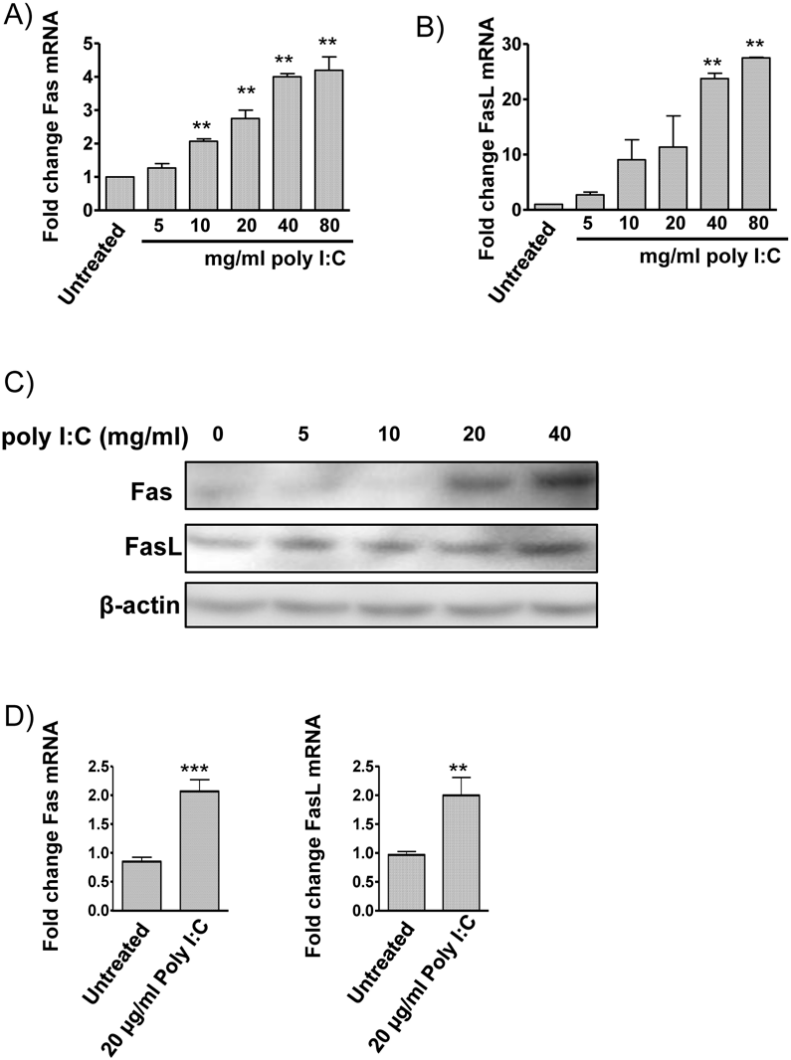
Stimulation of macrophages with poly I:C increases expression of Fas and FasL. THP-1 cells were differentiated with 100 μg/ml PMA for 72 hrs. Cells were treated with increasing concentrations of poly I:C, and Fas and FasL expression detected by qRT-PCR after 8 hrs (**A, B**) and Western blotting after 48 hrs (**C**). Human monocyte-derived macrophages from three separate donors were treated with poly I:C (20 μg/ml) and Fas and FasL expression detected by qRT-PCR after 8 hrs (**D**). Results shown are representative of three separate experiments. Values are shown as Mean ± SEM, (n = 3). ** p<0.01 and *** p<0.001.

Given that some viruses have been shown to affect Fas and FasL expression on macrophages [13, 14], we first investigated the ability of the dsRNA viral mimetic, poly I:C, to alter expression of Fas or FasL. THP-1-derived macrophages were stimulated with increasing concentrations of poly I:C and changes in Fas and FasL expression determined by both quantitative real-time PCR (qRT-PCR) and Western blotting. Treatment of THP-1-derived macrophages with poly I:C increased both Fas and FasL expression in a dose dependent manner at the mRNA (Fig 1A and 1B) and protein level (Fig 1C). To determine whether these results were specific to the THP-1 cell line, monocytes from healthy human donors were isolated and differentiated into macrophages (hMDM). Fas and FasL were also augmented in hMDMs following poly I:C stimulation (Fig 1D).

### Poly I:C-induced IFNβ, TNFα, IL-8 and IL-10 is reduced by activation of Fas, whilst IP-10 is augmented

**Fig 2:**
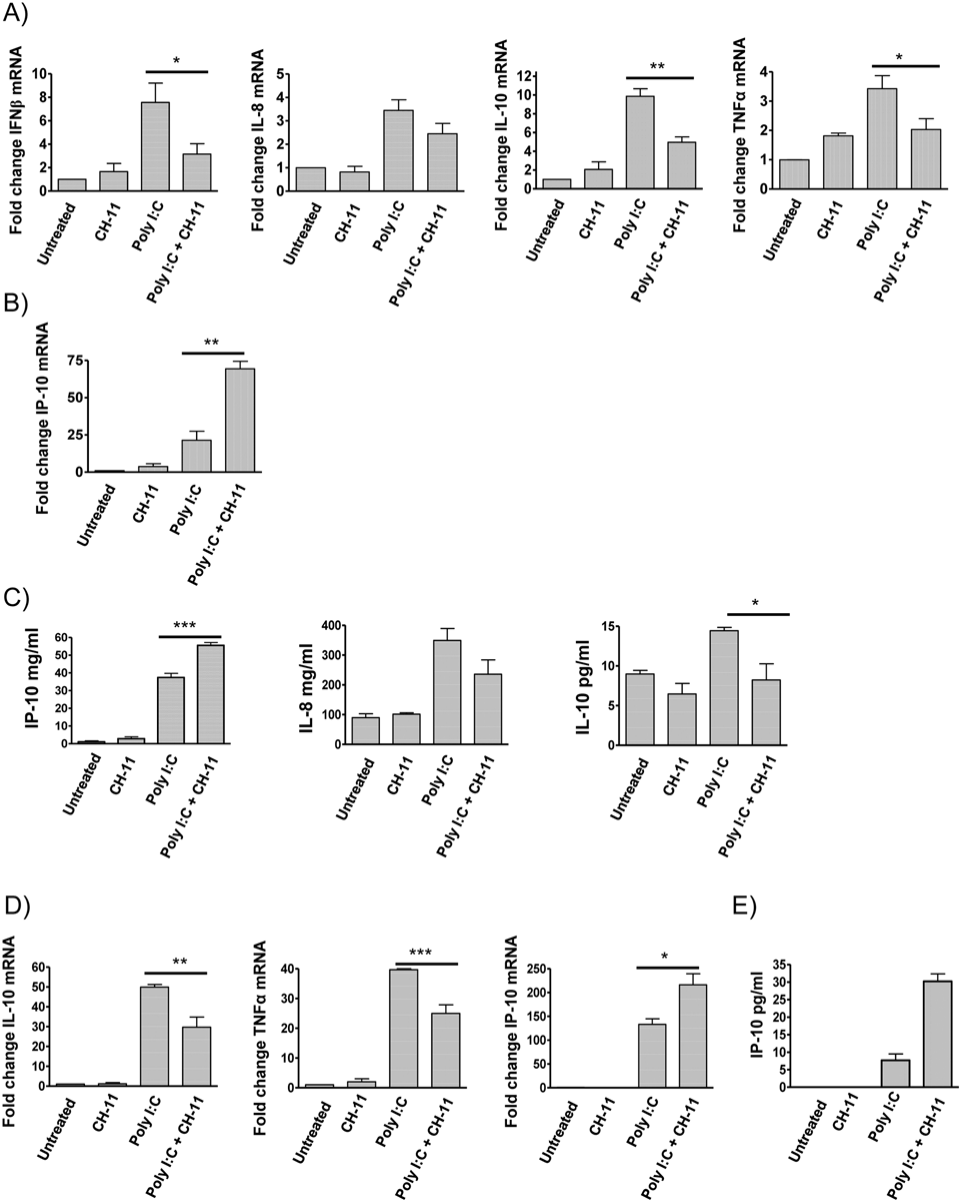
Activation of Fas decreases poly I:C-induced IL-8, IL-10, TNFα and IFNβ expression. THP-1-derived macrophages were treated with 100 ng/ml CH11 for 1 hr followed by stimulation with 20 μg/ml poly I:C. Changes in cytokine expression were detected by qRT-PCR after 8 hrs (**A, B**) and by ELISA after 48 hrs (**C**). Human monocyte-derived macrophages from three separate donors were treated with 100 ng/ml CH11 for 1 hr followed by stimulation with 20 μg/ml poly I:C. Changes in cytokine expression were detected by qRT-PCR after 8 hrs (**D**) and by ELISA for IP-10 after 48 hrs (**E**). Data shown are a combination of three independent experiments, with values shown as Mean ± SEM. * p<0.05, ** p<0.01 and *** p<0.001.

As expression of both Fas and FasL was increased by poly I:C stimulation, we next determined whether poly I:C-induced cytokine production was affected by Fas ligation. Stimulation of macrophages with poly I:C induced the transcription of IFNβ, IL-8, IL-10, TNFα, (Fig 2A) and IP-10 (Fig 2B). Stimulation of cells with the agonistic anti-Fas antibody, CH11, alone induced a very low increase in TNFα and IP-10 transcription. However, with the exception of IP-10, activation of Fas and subsequent stimulation with poly I:C significantly reduced poly I:C-induced expression of all cytokines (Fig 2A). In contrast, co-stimulation of cells with both poly I:C and CH11 significantly augmented expression of IP-10 (Fig 2B). Changes in poly I:C-induced IP-10, IL-8 and IL-10 production were confirmed at the protein level by ELISA (Fig 2C).

Changes in cytokine expression were also examined in hMDMs. Similar to the findings shown in Fig 2A, stimulation of Fas alone had no significant effect on cytokine production by hMDMs (Fig 2D). Fas activation, however, reduced poly I:C-induced cytokine production of both TNFα and IL-10, whilst IP-10 mRNA expression was augmented (Fig 2D). IP-10 was also augmented at the protein level (Fig 2E). In summary, these results indicate that activation of Fas signalling alters the balance between IP-10 and other inflammatory cytokines in macrophages in response to a dsRNA mimetic.

### Changes in poly I:C-induced cytokine production by activation of Fas occurred independently of Fas-mediated apoptosis

**Fig 3:**
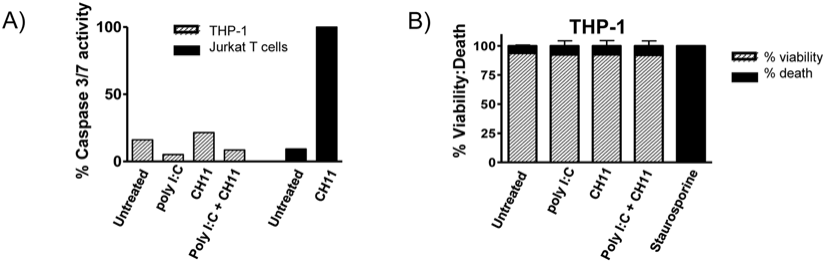
Fas activation in THP-1-derived macrophages does not induce caspase 3/7 activation. THP-1-derived macrophages and Jurkat T cells were treated with 100 ng/ml CH11 and/or 20 μg/ml poly I:C for 24 hrs, or with 5 μM staurosporine as indicated. Both caspase 3/7 activation (**A**) and cell viability (**B**) were determined after 24 hrs by fluorescence and trypan blue, respectively. Data shown are representative of three independent experiments, with values shown as Mean ± SEM.

Given the well characterised apoptotic function of Fas, we next investigated whether the altered cytokine production by THP-1 cells following CH11 stimulation was coupled to Fas-mediated apoptosis. Stimulation of THP-1 macrophages with either poly I:C or CH11 did not result in enhanced caspase 3/7 activation (Fig 3A). These results are consistent with previous studies, whereby activation of Fas in THP-1 cells did not trigger apoptotic cell death [8]. To confirm that CH11 was able to induce apoptosis in Fas-sensitive cells, Jurkat T cells were treated with CH11 which resulted in strong caspase 3/7 activation (Fig 3A). Cell viability of THP-1-derived macrophages was also unaffected by either poly I:C or CH11 treatment (Fig 3B). In contrast, staurosporine reduced the viability of the cells. Together these data indicate that the altered cytokine profile was not due to CH11- induced apoptosis.

### Increased IP-10 production in response to CH11 and poly I:C is not specific to either TLR3, RIG-1 or MDA5

**Fig 4:**
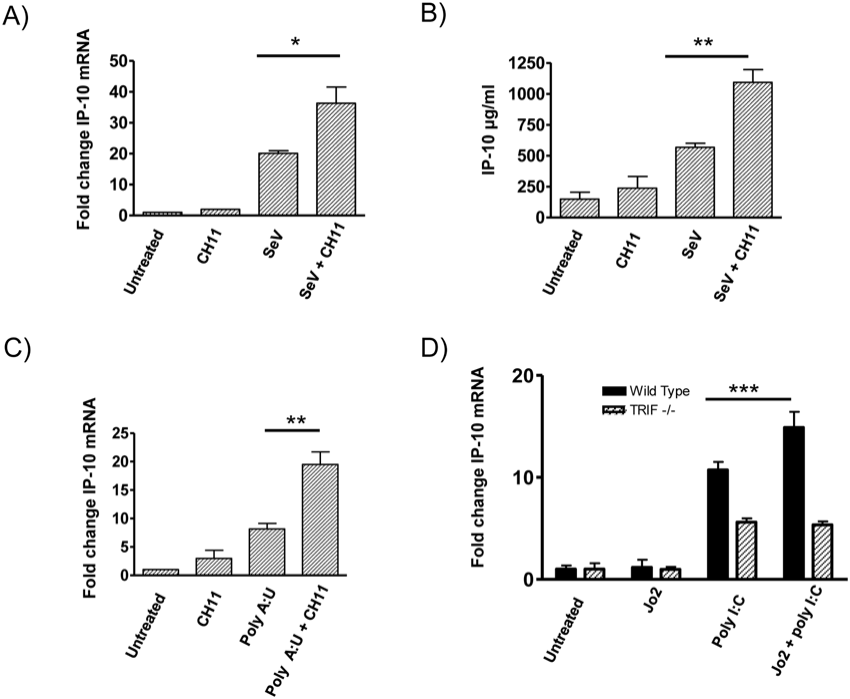
The augmentation of poly-I:C-induced IP-10 production following ligation of Fas is mediated by both TLR3 and RIG-I. THP-1-derived macrophages were treated with 100 ng/ml CH11 for 1 hr followed by infection with Sendai virus (**A,B**) or stimulation with poly A:U (**C**) for a further 8 hrs. Changes in IP-10 production were determined by qRT-PCR (**A,C**) or ELISA (**B**). Immortalised wild type and TRIF-^/-^ BMDMs were stimulated with 10 ng/ml murine agonistic Fas antibody (Jo2) for 1 hr followed by stimulation with 20 μg/ml poly I:C for a further 8 hrs, with changes in cytokine expression were detected by qRT-PCR (**D**). Data shown are a combination of three independent experiments, with values shown as Mean ± SEM. * p<0.05, ** p<0.01 and *** p<0.001.

dsRNA is known to be detected by several immune receptors including TLR3, RIG-I and MDA5. In order to investigate which of the poly I:C receptors were responsible for the augmented IP-10 production seen in response to stimulation with CH11 and poly I:C, THP-1 macrophages were infected with Sendai virus, which is detected by RIG-I [15] and/or MDA-5 [16], or were stimulated with poly A:U, which specifically activates TLR3. Stimulation of Fas with Sendai virus resulted in a 2 fold increase in IP-10 production relative to Sendai virus alone (Fig 4A and 4B). Similarly, Fas ligation significantly augmented poly A:U-induced IP-10 production (3 fold increase) (Fig 4C).

Given that TLR3 signals through TRIF, the involvement of the TLR3 signalling pathway was further investigated using TRIF knockout immortalised bone marrow-derived macrophages (TRIF-/- iBMDM). Consistent with our data using the human agonistic Fas antibody CH11 (Figs 3 and 4), stimulation of Fas with the murine agonistic Fas antibody, Jo2, significantly augmented poly I:C-induced IP-10 production by wild type iBMDMs (Fig 4D). In contrast, no augmentation of IP-10 production was seen in the TRIF-/- iBMDMs, indicating that the TLR3 and TRIF signalling pathways are involved in the ability of Fas to augment IP-10 production. This data indicates that the ability of Fas to augment poly I:C-induced IP-10 is not PRR specific.

### Co-stimulation with poly I:C and CH11 reduces poly I:C-induced phosphorylation of JNK and p38 MAPK

**Fig 5:**
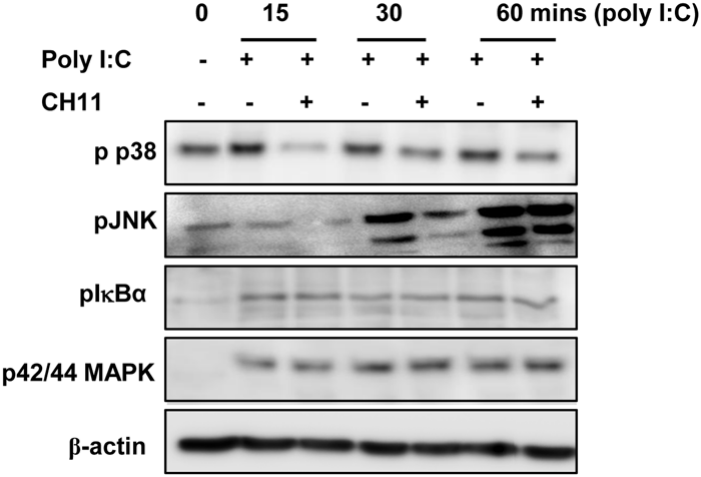
Fas activation suppresses poly I:C-induced activation of MAP Kinases p38 and JNK. THP-1 macrophages were treated with CH11 1 hr prior to poly I:C stimulation and Western blotting for phosphorylated p38 MAPK, JNK, IκBα and p42/p44 MAPK was performed. Levels of β-actin were assessed to ensure equal loading of protein. Data shown are representative of three independent experiments.

In order to determine how Fas activation augments poly I:C-induced IP-10, we next examined signalling pathways downstream of the receptors for poly I:C. The effect of CH11 on poly I:C-induced activation of NFκB and the MAP kinases (MAPK) p42/44, p38 and JNK were examined. Co-stimulation with both poly I:C and CH11 resulted in markedly reduced phosphorylation of the MAP kinases p38 and JNK at 15, 30 and 60 minutes of stimulation, relative to poly I:C alone. In contrast, phosphorylation of IκBα and p42/p44 MAPK was unaffected (Fig 5).

### The AP-1 transcription factor negatively regulates IP-10 production, with the Fas adaptor FADD activating AP-1

**Fig 6:**
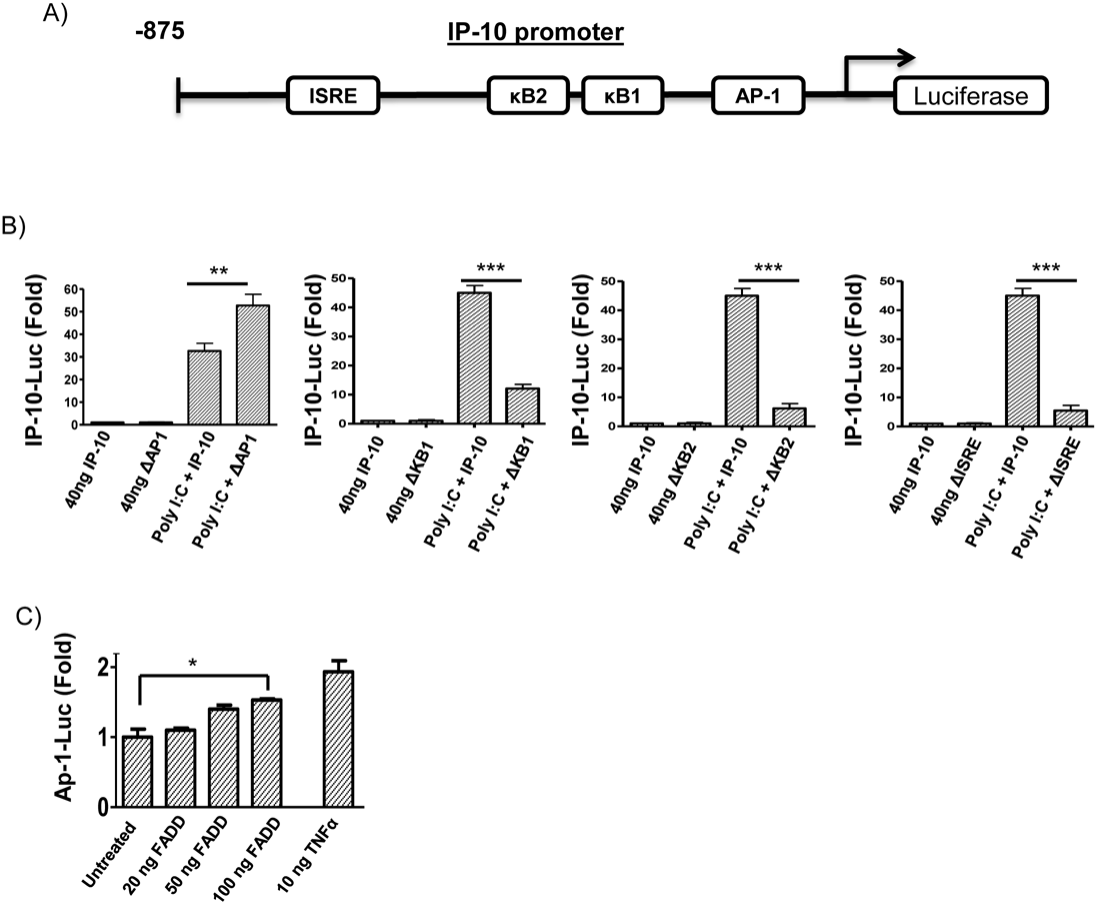
AP-1 negatively regulates the IP-10 promoter in response to poly I:C, with transfection of FADD augmenting AP-1 luciferase activity. HEK-293/TLR3 cells were transfected with full length IP-10 luciferase plasmids or IP-10 luciferase plasmids containing point deletions in binding sites for NF-κB (κB1, κB2), proximal ISRE or AP-1. A schematic of these putative binding sites is illustrated **(A)**. 24 hrs post transfection, cells were stimulated with 20 μg/ml poly I:C for 6 hours with IP-10 luciferase activity expressed as fold-change over TK-renilla activity **(B)**. Cells were transfected with an AP-1 luciferase reporter plasmid and the indicated doses of FADD plasmid. 24 hrs after transfection cells were stimulated with 20 μg/ml poly I:C or 10 ng/ml TNFα for a further 6 hrs. AP-1 activity were measured and expressed as fold-change over TK-renilla activity **(C)**. Data shown are a combination of three independent experiments, with values shown as Mean ± SEM.

As phosphorylation of JNK and p38 MAPK are known to result in activation of the transcription factor AP-1, this suggested that the ability of Fas to augment poly I:C-induced IP-10 was due to an effect on the AP-1 transcription factor. To investigate this, we first examined the IP-10 promoter using IP-10 promoter luciferase constructs mutated at the AP-1 binding site, two separate NFκB binding sites (κB2 and κB1) and the ISRE binding site (Fig 6A). Cells transfected with the IP-10 promoter mutated at the AP-1 binding site showed augmented IP-10 luciferase activity in response to poly I:C (Fig 6B). This augmentation parallels the increased IP-10 expression seen upon co-stimulation with CH11 and poly I:C (Fig 2). In contrast, poly I:C-induced IP-10 activation was reduced following mutation of two separate NFκB binding sites and also of the ISRE binding site (Fig 6B). These results indicate that the AP-1 transcription factor represses poly I:C-induced IP-10 production.

As FADD is the primary adaptor molecule for Fas, we next investigated whether transfection of cells with FADD altered activation of an AP-1 luciferase construct. Whilst all stimuli examined showed weak activation of the AP-1 luciferase, a low (1.5 fold), but consistent and dose dependant activation of AP-1 was seen upon transfection with FADD (Fig 6C), a level comparable to that seen with stimulation with TNFα (1.8 fold). This data strongly supports a role for Fas and FADD in the activation of the AP-1 transcription factor, thus suppressing poly I:C-induced IP-10 production. These data suggest that the reduction in poly I:C-induced p38 and JNK phosphorylation observed upon Fas stimulation reduces binding of AP-1 to the IP-10 promoter, alleviating AP-1-mediated repression of the promoter, thus augmenting poly I:C-induced IP-10 production.

### FADD suppresses poly I:C-induced IP-10

**Fig 7:**
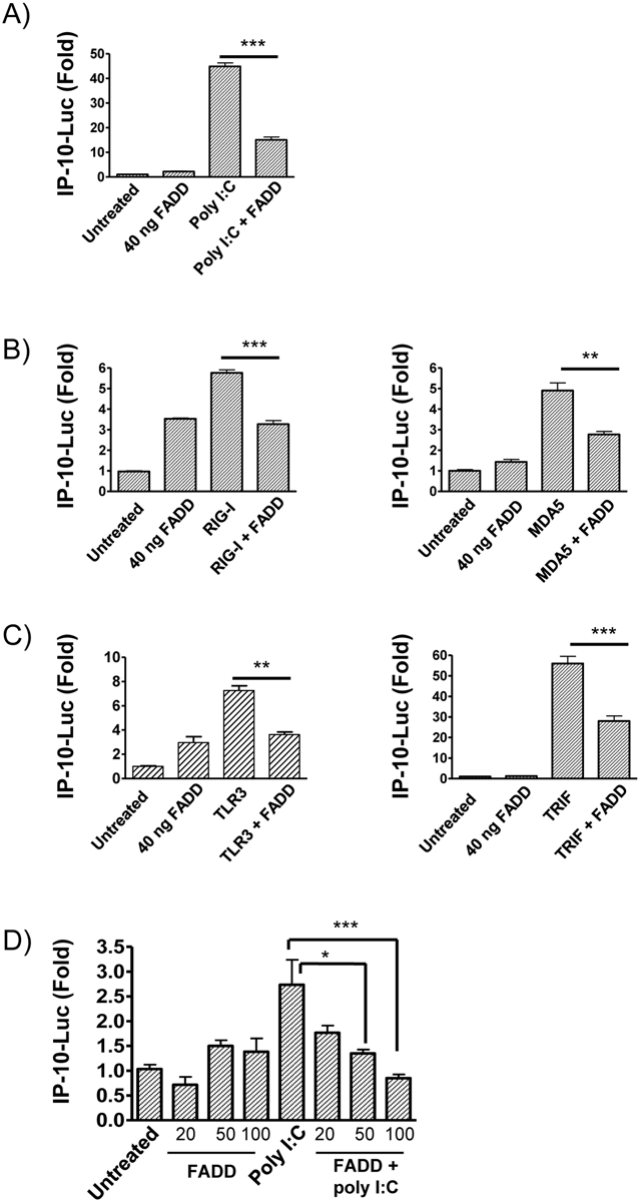
Overexpression of FADD inhibits poly I:C, TLR3, RIG-I, MDA5 and TRIF-induced IP-10. HEK293-TLR3 cells **(A)**, HEK293T cells **(B, C)** and RAW264.2 cells **(D)** were transfected with an IP-10 luciferase reporter plasmid and the indicated doses of FADD plasmid. Cells were also transfected with 40 ng of plasmid encoding RIG-1, MDA5, TLR3 or TRIF **(B, C)**. 24 hrs after transfection, cells were stimulated with 20 μg/ml poly I:C **(A,D)**. IP-10 activity was measured and expressed as fold-change over TK-renilla activity. Data shown are a combination of three independent experiments, with values shown as Mean ± SEM. * p<0.05, ** p<0.01 and *** p<0.001.

To further confirm a role for FADD in suppressing IP-10 production, HEK293 cells stably expressing TLR3 (HEK293-TLR3) were transfected with FADD and an IP-10 luciferase reporter construct. Overexpression of FADD suppressed poly I:C-induced IP-10 luciferase activity (Fig 7A). As stated previously, dsRNA is detected by TLR3, RIG-I and MDA5. In order to investigate whether FADD also suppresses RIG-I and MDA5-induced IP-10 luciferase activity, HEK293 cells without stable transfection of TLR3 were used. The use of these cells allowed us to negate any potential confounding effects of the TLR3 overexpression in the HEK293-TLR3 cell line. Plasmids encoding RIG-I and MDA5 were transfected into HEK293 cells, either with or without a plasmid encoding FADD. Consistent with a role for FADD in suppressing poly I:C-induced IP-10, overexpression of FADD reduced both RIG-I- and MDA5-induced IP-10 luciferase activity (Fig 7B).

Given that TLR3 signals through TRIF, we next investigated whether transfection of cells with FADD would also suppress TRIF-induced IP-10 luciferase activity. We initially confirmed that overexpression of FADD reduced TLR3-induced IP-10 luciferase activity in HEK293 cells (Fig 7C). Similarly overexpression of FADD also inhibited the ability of TRIF to activate the IP-10 luciferase construct (Fig 7C).

Finally, we confirmed a role for FADD in suppressing IP-10 production in macrophages using RAW264.7 cells, a murine macrophage cell line. RAW264.7 cells were transfected with FADD and an IP-10 luciferase reporter construct. Overexpression of FADD also suppressed poly I:C-induced IP-10 luciferase activity in a dose dependent manner in this cell type (Fig 7D).

Taken together, these data support a role for FADD in inhibiting poly I:C-induced IP-10, with this inhibition not specific for any of the receptors that detect poly I:C.

## Discussion

The findings of this study demonstrate that activation of Fas modulates poly I:C-induced inflammatory responses. Specifically we have shown that stimulation of macrophages with an agonistic Fas antibody prior to stimulation with poly I:C results in a reduction in IFNβ, IL-8, IL-10 and TNFα but an augmentation in IP-10 production. Fas ligation reduced poly I:C-induced phosphorylation of both JNK and p38 MAPK. Moreover, mutation of the AP-1 transcription factor binding site on the IP-10 promoter resulted in enhanced poly I:C-induced IP-10. The Fas adaptor protein FADD played a role in this process, with FADD activating AP-1, thus inhibiting poly I:C-induced IP-10. Together these data show that the ability of Fas to enhance poly I:C-induced IP-10 production may occur through modulating the activation of the AP-1 transcription factor.

Evidence for crosstalk between Fas and TLR signalling has been identified from several previous studies. In intestinal epithelial cells, signalling through Fas has been shown to augment TLR4- and TLR5-induced cytokine production [12]. Moreover, suppression of Fas signalling reduced LPS-induced cytokine production by macrophages [11]. To the best of our knowledge, no study to date has examined direct crosstalk between Fas and PRRs involved in the anti-viral immune response. Our findings demonstrate that activation of Fas modulates poly I:C-induced cytokine production in both a positive (IP-10) and a negative (TNFα, IL-8, IFN-β, IL-10) manner implicating a role for Fas ligation in tailoring the immune response following viral infection. Activation of innate immune pathways following viral infection leads to infiltration of pro-inflammatory immune effector cells such as T cells. Many of these are recruited in response to enhanced IP-10 production, with IP-10 playing an important role in both effector T cell generation and trafficking *in vivo* [4]. Our data indicate a viral dsRNA mimetic increases Fas and FasL expression on macrophages and that activation of Fas augments poly I:C- and Sendai virus-induced IP-10 production. As activated T cells also express FasL, it is possible that the Fas-FasL interaction between T cells and macrophages augments IP-10 production in response to viral infection. Consistent with our findings for a role for Fas in augmenting poly I:C-induced IP-10, mice lacking Fas or FasL showed delayed clearance of herpes simplex virus-2, with impaired immune cell recruitment [17, 18]. Indeed, some viruses, such as human herpes virus 8 and the poxvirus mollusc contagiosum virus, have been shown to target the Fas signalling pathway, protecting virally infected cells from Fas-mediated apoptosis [19, 20]. The findings from the current study suggest that blocking Fas signalling by viral proteins may also potentially interfere with the induction of an effective immune response to some viruses.

Increased levels of IP-10 have also been implicated in a variety of human diseases including infectious diseases, chronic inflammation, immune dysfunction, tumour development, metastasis and dissemination [3–5]. Since IP-10 plays a significant role in leukocyte homing to inflamed tissues, increased production of IP-10 may exacerbate inflammation and cause significant tissue damage [4–6]. It is important, therefore, that following viral infection, IP-10 levels are regulated to ensure an adequate immune response whilst avoiding an exacerbated inflammatory response. Thus, in situations of excessive T cell recruitment as characterised by many chronic inflammatory conditions, augmented IP-10 production by macrophages, as a result of Fas activation, may further exacerbate the inflammatory response.

**Fig 8:**
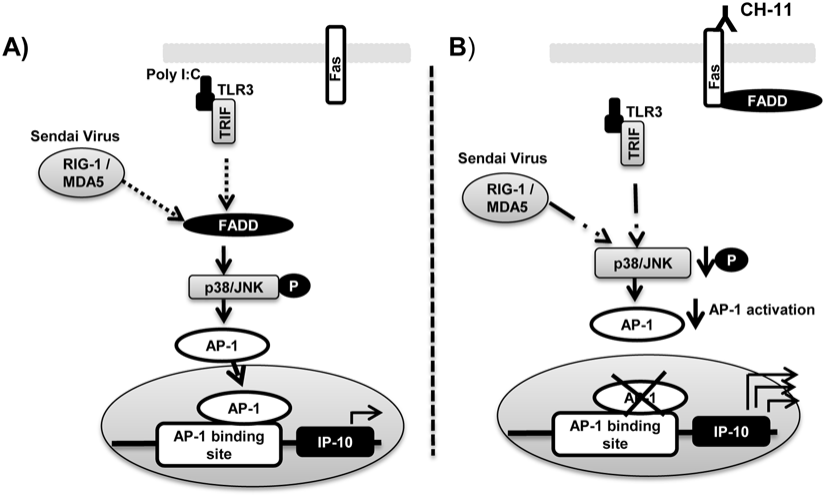
Schematic of mechanism whereby Fas activation may enhance poly I:C-induced IP-10. In the absence of Fas engagement, poly I:C, acting through both TLR3 and RIG-I, cause the phosphorylation of JNK and p38 MAPK in a FADD dependant manner. This results in AP-1 activation and translocation of AP-1 to the nucleus, where it acts to repress IP-10 production. Dashed lines indicate that the precise position of FADD in this pathway is unknown **(A)**. Upon Fas activation, FADD is recruited to Fas, reducing poly I:C-induced JNK and p38 MAPK activation. This reduces the levels of AP-1, thereby alleviating AP-1-mediated repression of the IP-10 promoter, resulting in enhanced IP-10 production. **(B)**

As Fas augmented both poly A:U and Sendai virus-induced IP-10, this indicated that the modulation of IP-10 was not specific to either the TLR3, MDA5 or RIG-I signalling pathways. Consistent with this, stimulation of Fas reduced the ability of poly I:C to induce the phosphorylation of the MAP kinases, JNK and p38. JNK and p38 MAPK are known to be involved in the activation of the AP-1 transcription factor. Mutation of the AP-1 binding site, in turn, augmented IP-10 transcription, indicating that AP-1 inhibits the IP-10 promoter. Thus, the ability of CH11 to reduce poly I:C-induced JNK and p38 MAPK may result in a reduction in the level of AP-1 binding to the IP-10 promoter, thus alleviating AP-1-mediated repression of IP-10 (illustrated in Fig 8). A similar inhibitory role for AP-1 on the IP-10 promoter was recently shown in hepatocytes following Hepatitis C (HCV) infection [9]. Although this study did not investigate the role of Fas during HCV infection, hepatocytes are known to express Fas [21]. Our data indicates that activation of Fas signalling in hepatocytes during HCV infection may play an important role in the induction of IP-10 in these cells.

We have also shown a role for the Fas adaptor, FADD, in the modulation of poly I:C-induced IP-10. FADD activated AP-1 and overexpression of FADD inhibited poly I:C-induced IP-10 production. Studies investigating the role of FADD in poly I:C signalling have shown that FADD is a critical signalling component of the RIG-I signalling pathway [22, 23]. FADD^-/-^ MEFs were defective in the production of type1 (α/β) IFNs in response to intracellular dsRNA [22]. FADD was also shown to be required for IRF7 induction through RIG-I, MDA5 and IPS-1 [23]. Similarly FADD^-/-^ MEF were also shown to be defective in poly I:C-induced IL-6 production [24]. Evidence for an alternative role for FADD in the anti-viral immune response comes from a recent study which identified an interaction between FADD and TRIM21. In this study, loss of FADD and TRIM21 led to higher levels of IFNα induction in response to Sendai virus. These authors concluded that FADD is inhibitory to viral-induced IFNα production [25]. The modulation of cytokine expression that we observed upon Fas ligation is consistent with an essential role for FADD in anti-viral signalling [22, 23]. FADD has been shown to be recruited to Fas upon Fas ligation with a consequent reduction in the level of FADD in the cytoplasm [26, 27], thereby potentially reducing the levels of FADD available to transduce anti-viral signalling pathways. This would also account for the reduction in levels of poly I:C-induced TNFα, IL-8, IL-10 and IFN-β that we observed upon Fas ligation. Interestingly, a direct interaction between Fas and TLR3, upon poly I:C stimulation, has recently been shown in microglial cells, with this interaction attenuating Fas-mediated apoptosis [28]. Whilst these authors did not examine the effect of this interaction on TLR3 signalling, it is possible that, should such an interaction also exist in macrophages, this may attenuate TLR3-mediated inflammation, possibly providing an alternative explanation for the ability of CH11 to downregulate poly I:C-induced cytokine responses, as observed in our study. Further work is required to examine whether such an interaction exists in macrophages and also to examine whether Fas interacts directly with the other receptors for poly I:C, namely RIG-I and MDA5.

Using overexpression studies we have shown that FADD inhibits RIG-I, MDA5, TLR-3 and TRIF-induced IP-10 production. Our data showing that overexpression of FADD inhibits TRIF-induced IP-10 production implies that FADD inhibits downstream of TLR3 and TRIF, possibly through a TRIF-FADD interaction. Indeed, FADD has been shown to interact with TRIF in a complex containing FADD/FLIP/RIP-1 in HeLa cells [29]. [29]. It is possible that this complex also exists in macrophages and that FADD exerts its effects on TLR3 signalling through this interaction. We have also shown a role for FADD in inhibiting RIG-I and MDA5- induced IP-10 production. FADD has been shown to be in a complex with IPS-1 (the adaptor for RIG-I and MDA5) and RIP-1 [30], and this interaction may be responsible for the modulation of RIG-I and MDA5- induced IP-10.

In conclusion, we demonstrate here a role for Fas in modulating poly I:C-induced cytokine production. These studies show that ligation of Fas specifically enhances IP-10 through modulation of p38 and JNK MAP kinases and the AP-1 transcription factor. It is not clear why AP-1 negatively regulates IP-10 and not the other cytokines examined here. This study points to a level of complexity present in the IP-10 promoter whereby the levels of IP-10 are tightly regulated in the anti-viral immune response. It may be that the requirement for a second activating signal, such as Fas ligation, ensures that the promoter is optimally induced only during active infection.

## Materials and Methods

### Reagents

Poly I:C and poly A:U were both obtained from Sigma (St. Louis, Missouri, USA). The human agonistic anti-Fas antibody CH11 was obtained from Merck-Millipore, (Billerica, MA, USA), with murine agonistic anti-Fas antibody Jo2 obtained from BD Biosciences (San Jose, CA, USA). Staurosporine was obtained from Sigma.

### Expression Plasmids/Reporter Constructs

The IP-10/CXCL-10 luciferase reporter plasmid and related mutants were obtained from Prof. David Proud (University of Calgary, Alberta, Canada) and are as described [31]. The FADD plasmid was obtained from Addgene (Addgene plasmid 31814) [24]. pcDNA3, TRIF, TLR3, and TK-Renilla plasmids were all kind gifts from Prof. Luke O’Neill, Trinity College Dublin, Ireland and are as described [32, 33]. The 3xAP-1pGL3 was obtained from Addgene via A. Dent. (Addgene plasmid 40342). The RIG-I and MDA5 plasmids were originally generated by Professor Takashi Fujita [34].

### Cell culture

THP-1 monocytes and Jurkat T cells (ECACC, Salisbury, United Kingdom) were maintained in RPMI-1640 media supplemented with 10% foetal calf serum (FCS) and 1% penicillin/streptomycin (P/S). THP-1 cells were differentiated into macrophages by treating cells with 100 μg/ml phorbol 12-myristate 13-acetate (PMA) (Sigma) for 72 hrs. HEK 293 cells and RAW264.7 macrophages (ECACC, Salisbury, United Kingdom) were maintained in DMEM supplemented with 10% FCS, 1% P/S. HEK-293/TLR-3 cells (Invivogen, Toulouse, France) were maintained in DMEM supplemented with 10% FCS, 1% P/S, 10 μg/ml blasticidin (Invivogen) and 100 μg/ml normocin (Invivogen). Immortalised wild-type and TRIF-/- bone marrow derived macrophages (iBMDMs), a kind gift from Dr. Sinead Miggin (Immune Signalling Laboratory, NUI Maynooth, Co. Kildare, Ireland), were maintained in DMEM containing 10% FCS and 1% P/S.

### Isolation of Human Monocyte-derived Macrophages

Blood samples were collected from healthy volunteers and monocytes extracted using Histopaque-1077 (Sigma). Mononuclear cells were seeded in 24 well plates and allowed to adhere for 7 days. Non-adherent cells were removed by washing. Adhered cells were assessed both visually and by CD68 immunostaining to ensure differentiation into macrophages. Ethical approval was obtained from Clinical Research Ethics committee of the Cork Teaching Hospitals. Ref no. ECM3 (p) 03/09/13. Informed written consent was obtained from each healthy volunteer.

### Sendai Virus (SeV) Infection

Sendai virus was supplied at 10(7.5)CEID[50]/0.2 mL (ATCC, Manassas, VA, USA). Initial dilutions of SeV were performed, with a final dilution of 1:10,000 in 1ml RPMI-1640 supplemented with 1% P/S and 10% FCS selected. Cells were infected for a total of 8 hrs.

### Western Blotting

Cells were treated with increasing concentrations of poly I:C as indicated in the figure legends and lysed directly in sample buffer (0.5M Tris, pH 6.8, sodium dodecyl sulphate, bromophenol blue, glycerol, deionised water, 100mM DL-Dithiothreitol). Samples were separated on a 10% SDS-polyacrylamide gel and transferred to Immobilon-P Transfer Membrane (Merck Millipore, Billerica, MA, USA). Membranes were probed overnight at 4°C with anti-Fas (Santa Cruz Biotechnologies, CA, USA), anti-FasL, (BD Biosciences, NJ, USA), anti-β-actin (Sigma), anti-phospho-IκBα, anti-phospho-p42/44 MAPK, anti-phospho-p38 MAPK and anti-phospho-JNK specific antibody (Cell Signaling, Danvers, MA, USA). Following incubation with the appropriate secondary antibodies, membranes were developed using chemiluminescent HRP substrate (Merck-Millipore).

### Quantitative Real Time PCR (qRT-PCR)

Total cellular RNA was isolated using the GenElute Mammalian Total RNA Mini kit (Sigma) according to the manufacturer’s instructions. cDNA was synthesised using the Tetro cDNA synthesis kit (Bioline, London, UK). RT-PCR was performed using an Applied Biosystems PRISM 7500 PCR system (Applied Biosystems, Life Technologies, Carlsbad, CA, USA) and TaqMan Gene Expression Master Mix. The following gene expression Taqman primer-probe sets (Applied Biosystems) were used: Fas, Hs00236330_m1; FasL, Hs00181225_m1; IL-8, Hs99999034_ml; TNFα, Hs00174128_m1; IFNβ, Hs00277188_s1; IL-10, Hs00961622; IP-10, Hs00171042_m1; GAPDH, 4352934E; IP-10, Mm00445235_m1; β-actin, 4352341E). All samples were run in triplicate and relative quantitation calculated using the 2-^ΔΔ^Ct method. Transcript levels were normalised to the amount of GAPDH or β-actin mRNA and expression levels shown as fold induction relative to untreated.

### Caspase 3/7 Assay

Cells were seeded overnight in black flat-bottomed 96-well plates at a density of 20,000 cells/well, treated with CH11 for 1 hr and subsequently with 20 μg/ml poly I:C for 24 hr, or were treated with each agonist separately. Apo-ONE caspase-3/7 reagent was added and following 1 hr incubation, fluorescence (485 excitation, 530 emission) was measured using a GENios Microplate Reader (Tecan Group Ltd, Männedorf, Switzerland). Changes in caspase 3/7 activation were normalised relative to untreated cells.

### Viability Assay

Cells were untreated or were stimulated with poly I:C, poly A:U, and/or CH11 as indicated in the figure legends. Cells were trypsinised and cell viability assessed using trypan blue.

### Luciferase assays

Transient transfections of HEK-293/TLR-3, HEK293 and RAW264.7 cells were performed using GeneJuice (Merck-Millipore) according to manufacturer’s instructions. Stimulations involving poly I:C were performed for 6 hrs prior to lysis of cells. Cells were lysed in Passive Lysis Buffer (10mM EDTA, 100mM DTT, 50% glycerol, 5% Triton X-100, 125mM Tris base, pH 7.8). Luminescent activity was then measured on Promega GloMax system (Madison, WI, USA).

### ELISA/MSD

Supernatant from THP-1 macrophages and hMDMs were stimulated with poly I:C and/or CH11 for 48 hrs. IL-8 and IL-10 were measured by ELISA (BioLegend, San Diego, California). IP-10/CXCL-10 protein analysis was performed using MSD plates (Meso-Scale Discover, Rockville, MD, USA) as per the manufacturer’s instructions.

### Statistics

Experiments were performed a minimum of three times in triplicate. All results, with the exception of the MEF experiments were statistically evaluated using One-Way Anova with Tukeys post-test. MEF experiments were analysed using Two-Way Anova, followed by a subsequent student t-test on the individual stimulations. Values of p<0.001 are indicated by three asterisks (***). Values of p < 0.01 are indicated by two asterisks (**). Values of p < 0.05 are indicated by one asterisk (*).
